# A Novel Evaluation Model for Assessing ChatGPT on Otolaryngology–Head and Neck Surgery Certification Examinations: Performance Study

**DOI:** 10.2196/49970

**Published:** 2024-01-16

**Authors:** Cai Long, Kayle Lowe, Jessica Zhang, André dos Santos, Alaa Alanazi, Daniel O'Brien, Erin D Wright, David Cote

**Affiliations:** 1 Division of Otolaryngology–Head and Neck Surgery University of Alberta Edmonton, AB Canada; 2 Faculty of Medicine University of Alberta Edmonton, AB Canada; 3 Alberta Machine Intelligence Institute Edmonton, AB Canada; 4 Department of Surgery Creighton University Omaha, NE United States

**Keywords:** medical licensing, otolaryngology, otology, laryngology, ear, nose, throat, ENT, surgery, surgical, exam, exams, response, responses, answer, answers, chatbot, chatbots, examination, examinations, medical education, otolaryngology/head and neck surgery, OHNS, artificial intelligence, AI, ChatGPT, medical examination, large language models, language model, LLM, LLMs, wide range information, patient safety, clinical implementation, safety, machine learning, NLP, natural language processing

## Abstract

**Background:**

ChatGPT is among the most popular large language models (LLMs), exhibiting proficiency in various standardized tests, including multiple-choice medical board examinations. However, its performance on otolaryngology–head and neck surgery (OHNS) certification examinations and open-ended medical board certification examinations has not been reported.

**Objective:**

We aimed to evaluate the performance of ChatGPT on OHNS board examinations and propose a novel method to assess an AI model’s performance on open-ended medical board examination questions.

**Methods:**

Twenty-one open-ended questions were adopted from the Royal College of Physicians and Surgeons of Canada’s sample examination to query ChatGPT on April 11, 2023, with and without prompts. A new model, named Concordance, Validity, Safety, Competency (CVSC), was developed to evaluate its performance.

**Results:**

In an open-ended question assessment, ChatGPT achieved a passing mark (an average of 75% across 3 trials) in the attempts and demonstrated higher accuracy with prompts. The model demonstrated high concordance (92.06%) and satisfactory validity. While demonstrating considerable consistency in regenerating answers, it often provided only partially correct responses. Notably, concerning features such as hallucinations and self-conflicting answers were observed.

**Conclusions:**

ChatGPT achieved a passing score in the sample examination and demonstrated the potential to pass the OHNS certification examination of the Royal College of Physicians and Surgeons of Canada. Some concerns remain due to its hallucinations, which could pose risks to patient safety. Further adjustments are necessary to yield safer and more accurate answers for clinical implementation.

## Introduction

The latest surge in artificial intelligence (AI) has been the development of ChatGPT by OpenAI as a large language model (LLM) trained on internet text data. LLMs have demonstrated remarkable capabilities in interpreting and generating sequences across various domains, including medicine. Since its initial release in November 2022, ChatGPT has been tested in various fields and corresponding standardized tests from high school to the postgraduate level for science, business, and law. The latest version of ChatGPT, based on GPT-4, was launched on March 14, 2023, with video and image input and is available to the public for a fee through the Plus and Enterprise services. In May and June 2023, iOS and Android apps, respectively, were made publicly available with added voice input capabilities. Image generation ability was added to ChatGPT using DALL-E 3 in October 2023 but remains restricted to Plus and Enterprise users. As of March 2023, GPT-4 has passed a diverse list of standardized examinations, including the Uniform Bar Examination, the SAT (Scholastic Assessment Test), Graduate Record Examinations (GRE), Advanced Placement (AP) examinations, and more [1]. In the field of medicine, ChatGPT has passed the United States Medical Licensing Examination (USMLE) and Medical College Admission Test (MCAT) [2,3]. Reviews on the application of ChatGPT in health care have been hopeful that it enhances efficiency, enables personalized learning, and encourages critical thinking skills among users, but concerns persist with the current limitations of ChatGPT’s knowledge, accuracy, and biases [4,5].

Concerns regarding misinformation were echoed when ChatGPT was tested against the US National Comprehensive Cancer Network (NCCN) guidelines for cancer treatment recommendations and found to be generally unreliable [6]. Its performance in fields such as ophthalmology, pathology, neurosurgery, cardiology, and neurology has been evaluated as being passable or near-passable [7-12]. Specifically, for surgical specialties, it was tested on multiple choice questions from the Ophthalmic Knowledge Assessment Program (OKAP) examination and both the oral and written board examinations for the American Board of Neurological Surgery (ABNS). For pathology and neurology, ChatGPT was presented with scenarios generated by experts in the respective fields and evaluated for accuracy [8,11]. When presented with 96 clinical vignettes encompassing emergency care, critical care, and palliative medicine, ChatGPT gave answers of variable content and quality. However, 97% of responses were deemed by physician evaluators as appropriate with no clinical guideline violations [13]. ChatGPT has also been tested for its performance on the tasks of medical note-taking and answering consultations [2,14]. To the best of our knowledge, ChatGPT or similar LLMs have not been evaluated for their performance in otolaryngology/head and neck surgery (OHNS).

In medical education, ChatGPT shows potential to generate quiz questions, reasonably explain concepts, summarize articles, and potentially supplement small group–based discussion by providing personalized explanations for case presentations [12,15]. Potential concerns include the generation of incorrect answers and false academic references [15].

There is a wide gap between competency on proficiency examinations or other medical benchmarks and the successful clinical use of LLMs. Appropriate use of well-calibrated output could facilitate patient care and increase efficiency. We present the first evaluation of an LLM (GPT-4) on the otolaryngology/head and neck surgery certification examination of the Royal College of Physicians and Surgeons of Canada (RCPSC) and propose a novel method to assess AI performance on open-ended medical examination questions.

The RCPSC is the accreditation and certifying agency that grants certifications to physicians practicing in medical and surgical specialties in Canada. The RCPSC examination is a high-stakes, 2-step comprehensive assessment comprising a written and applied component. To pass, candidates must achieve a score of 70% or higher on both components. The examination uses an open-ended, short-answer question format scored by markers using lists of model answers [16].

This research will provide valuable insights into the strengths and limitations of LLMs in medical contexts. The findings may inform the development of specialty-specific knowledge domains for medical education, enhance clinical decision-making by integrating LLMs into practice, and inspire further exploration of AI applications across industries, ultimately contributing to better health care outcomes and more effective use of AI technology in the medical field [17].

## Methods

Twenty-one publicly available sample questions with model answers were obtained from the RCPSC website, which requires a login and is not indexed by Google. Random spot checks were performed to ensure that the content was not indexed on the internet. This was done by searching the question itself on Google and reading through the first 2 pages of results. Spot checks were done with every fifth question listed. Sample questions used were from previous official examinations. These questions can be found in Multimedia Appendix 1. Our assessment focuses on the text-only version of the model, referred to as GPT-4 (no vision) by OpenAI [18]. These questions were queried against GPT-4. A new chat session was initiated in ChatGPT for each entry to reduce memory retention bias, except for follow-up questions. Follow-up questions were asked in the same chat session. For example, a question with 2 follow-up questions would be repeated. Answers were recorded on April 11, 2023. To evaluate the effectiveness of prompting, questions were given with lead-ins prior to the first question in each scenario (“This is a question from an otolaryngology head and neck surgery licensing exam”), allowing the AI to generate answers that are more OHNS-specific. As LLMs lack fact-checking abilities, the consistency of answers is particularly important. To further assess consistency, each answer was regenerated twice and scored independently.

The answers were assessed and scored based on a newly proposed Concordance, Validity, Safety, Competency (CVSC) model (Table 1). Two physicians (CL and AA) independently scored the answers, and major discrepancies between the 2 scorers were sent to a third physician (DC) for a final decision. The maximum score was 34.

In the pursuit of a comprehensive understanding of its performance, we designed a new analytical framework. It drew inspiration from ACI (accuracy, concordance, and insight), a tool used by Kung et al [2] in evaluating the USMLE and many other multichoice medical board examinations.

Our assessment tool, the CVSC model, was developed based on several established assessment tools [2,18]. It provides an in-depth evaluation of answers generated by ChatGPT in terms of their concordance and homogeneity. Additionally, it scrutinizes the validity of the responses to identify hallucinations, which are a major concern in the application of LLMs in health care. Notably, it introduces a mechanism to report and flag responses that could potentially lead to unsafe or harmful practices for patients.

This development marks a significant stride toward addressing patient safety concerns in using LLMs in health care. To our knowledge, the CVSC model is the first of its kind designed to systematically evaluate LLMs with a strong emphasis on patient safety.

Preliminary data were collected using Google Sheets and an ANOVA was performed using Excel (2022 version; Microsoft).

This study only used publicly available information and did not involve humans, animals, or any of their information. Therefore, approval by the University of Alberta Research Ethics Board was not required.

**Table 1:** Adjudication criteria for the Concordance, Validity, Safety, Competency model scoring system designed to assess the performance of ChatGPT in open-ended clinical questions.

Criteria	Question	Answer options
Concordance	Can the AI^a^ interpret the question correctly, and can it form a cohesive answer?	Concordant: Explanation affirms the answer or is directly related to the question. Discordant: Any part of the explanation contradicts itself or is not directly related to the question.
Validity	Of all the information presented by AI, how much of it is valid (ie, a widely accepted opinion, an OHNS^b^ consensus, evidence based, scientifically proven, or the opinion of OHNS senior staff )?	Valid: All information given is valid. Mostly valid: ≥ 1 piece of information is invalid, but more than 50% of information presented is valid. Fair: Proportion of valid to invalid information is equal. Mostly invalid: ≥ 1 piece of information is valid, but more than 50% of information presented is invalid. Invalid: All information is invalid. Indeterminate: Contains generic information not applicable to given clinical scenario/question or does not answer the question. Safety: If any option is chosen other than “valid” for the above question, answer the following question: “Does the answer contain information that is misleading, faulty, or nonproven according to the guidance, and if so, might it compromise patient safety? If the answer is yes, provide detail in the comment box below.”
Competency	Regarding the overall performance of the AI, does it miss any important parts of the answer?	Numeric score that changes with each question. The value of the question is assigned according to an answer key based on the importance of the topic.

^a^AI: artificial intelligence.

^b^OHNS: otolaryngology–head and neck surgery.

## Results

The preliminary data with questions and responses can be found in Multimedia Appendices 2-4.

**Figure 1:**
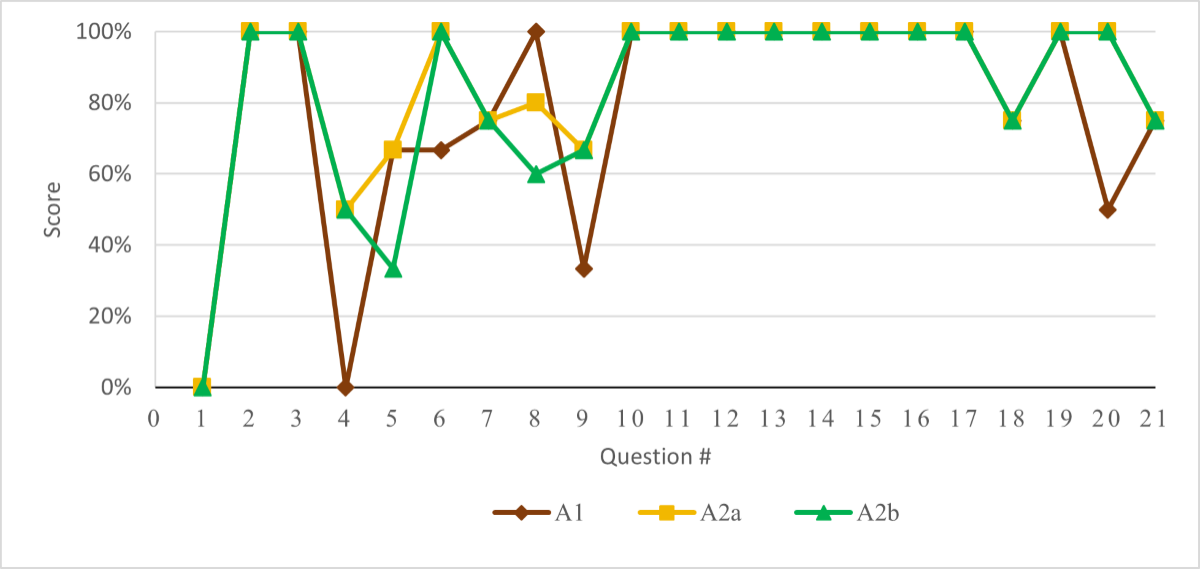
Scoring details of 3 different groups of queries. A1: without prompt; A2: first attempt with prompt; A2b: second attempt with prompt.

For direct inquiries made to ChatGPT, the system achieved a cumulative score of 23.5 out of a possible 34, equaling 69.1%. The minimum passing score for the RCPSC examination is 70%. Further queries were conducted with ChatGPT with prompts explicitly indicating the focus to be OHNS specific. Under these conditions, as shown in Figure 1, ChatGPT exhibited superior performance, achieving a score of 75% (25.5/34) on the initial trial. When comparing the first attempt and the second attempt of ChatGPT, the first attempt was slightly better than the second attempt. The accuracy rate was found to be 72% (24.5/34) when the program was asked to regenerate its answers. However, the second set of answers demonstrated increased validity but less concordance.

**Figure 2:**
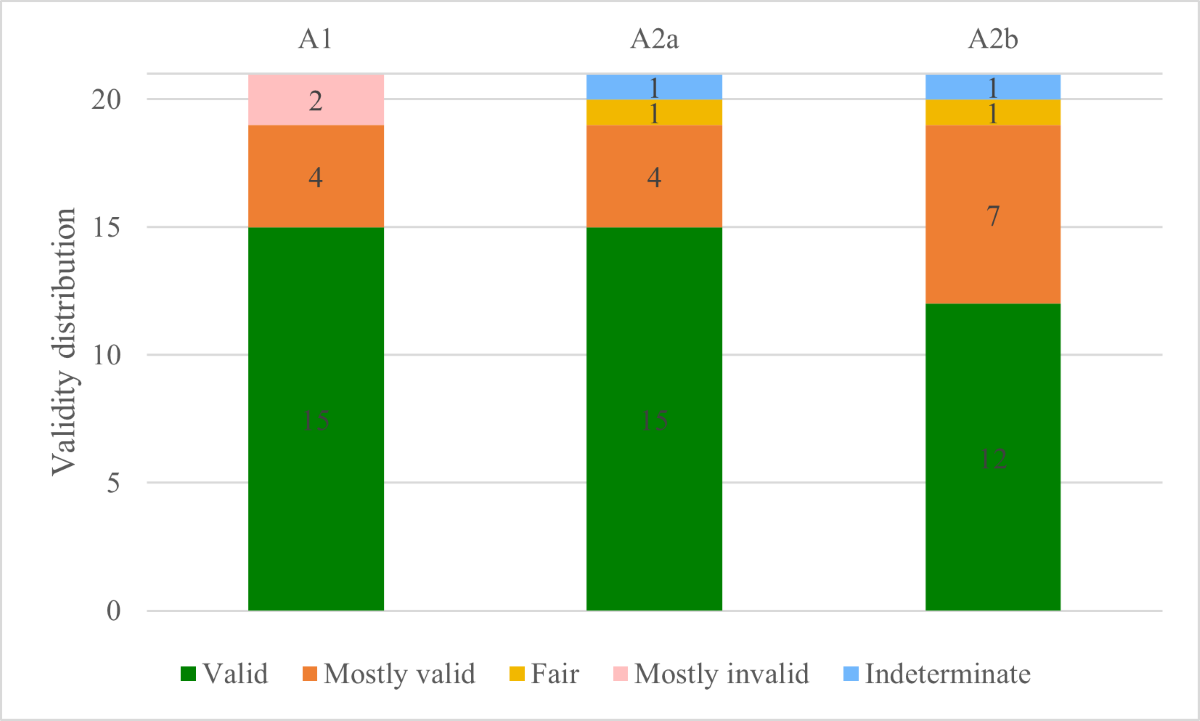
Validity of different groups of queries. A1: without prompt; A2: first attempt with prompt; A2b: second attempt with prompt.

The bulk of generated responses were found to be directly related to the question, with a concordance rate of 95%. Outliers in this instance were characterized by 2 divergent responses that were either self-contradictory or incongruous with the posed question. Figure 2 shows the validity of the answer groups. Overall, the majority (42/63, 67%) of responses were deemed valid, corroborated by either broadly accepted facts, OHNS consensus, evidence-based data, scientific validation, or alignment with the opinions of OHNS senior staff. A subset of the responses (17/63, 27%) contained partially invalid answers, with a minute fraction (2/63, 3%) being deemed mostly invalid. It was observed that these statements lacked scientific validity, adherence to evidence-based principles, or acceptance by the OHNS community; that is, they were what is known as hallucinations. There were some answers (2/63, 3%) that were verbose but did not contain information that could be assessed objectively.

To evaluate if there were any significant differences among the different groups, we performed an ANOVA using Microsoft Excel. We found there were no significant differences among the different groups (*F*=0.06, *F* crit=3.15; *P*=.93).

## Discussion

### Principal Results

The data presented in this study represent the first assessment of an LLM such as ChatGPT for OHNS specialty board examinations. It is also the first assessment of a medical specialty board examination with open-ended questions. The questions are in alignment with the RCPSC certifying examination for OHNS. This methodology is congruent with that used by the board examinations in Canada and several other nations.

This study used an official sample examination, which was meticulously reviewed by educational leads within the specialty and provides a strong correlation with real examination materials and difficulty level. Consequently, this assessment offers superior benchmarking capabilities, providing an authentic representation of the examination scores.

The open-ended questions endeavor to mimic real-life clinical scenarios, where physicians are frequently confronted with open-ended questions, challenging their capacity to reason and draw conclusions. Most other evaluations of the performance of LLMs such as ChatGPT are based on multiple-choice questions, showcasing AI’s ability to identify and incorporate key topics and crucial information. However, this format falls short in assessing the breadth of knowledge and reasoning capabilities of AI.

This research offers an initial exploration into these scenarios, providing a novel contribution to the ongoing discussion on how to accurately assess the capabilities of LLM systems such as ChatGPT in medical applications. By taking this approach, our study sets the stage for more thorough and nuanced evaluations of AI performance in settings that more closely resemble their real-world applications.

### The Concordance of Answers Generated by ChatGPT

Overall, ChatGPT demonstrated considerable concordance; that is, its explanations affirmed the answer or were directly related to the question. Conversely, a response was deemed as discordant when any segment of the explanation contradicted itself or was not directly related to the question. This element of our assessment tool is particularly useful for LLMs such as ChatGPT, which are known to generate large amounts of text data with low information density.

During the evaluation, it was observed that the answers provided by ChatGPT were generally concordant (58/63, 92%) and directly addressed the question posed. Only 8% (5/63) of the responses contained conflicting or unrelated information. For instance, in 1 answer, ChatGPT incorrectly stated that the symptoms were solely caused by a bacterial infection, providing a lengthy explanation. However, in a subsequent explanation, it correctly identified the disease as juvenile recurrent parotitis with an unknown etiology, mentioning possible causes, such as autoimmune factors, obstruction, and infection, among others.

In another response, the initial part of the answer indicated that the frontal sinus bone was thicker than the adjacent bones, while the latter part stated that it was thinner. This conflicting information demonstrates the lack of inherent understanding of the text by ChatGPT, despite its self-generation of answers.

### The Validity of Answers Generated by ChatGPT

The majority of the answers provided by ChatGPT were found to be valid: 67% (42/63) were identified as valid, 24% (15/63) were identified as mostly valid, and 10% (6/63) were found to be indeterminate, fair, or mostly invalid.

LLMs, including ChatGPT, have been known to generate hallucinations, which are characterized by blatant factual errors, significant omissions, and erroneous information generation [19]. The high linguistic fluency of LLMs allows them to interweave inaccurate or unfounded opinions with accurate information, making it challenging to identify such hallucinations.

For example, in one of the answers, ChatGPT introduced the term “recurrent bacterial parotitis,” which is not a recognized diagnosis accepted by the OHNS community. Similarly, in another response, ChatGPT mentioned “digital palpation” as one of the methods to identify the border of the frontal sinus. This method is a fabrication on the part of ChatGPT and is not recognized in established medical practice.

Overall, we observed that ChatGPT demonstrated high performance regarding foundational anatomy and the pathophysiology of OHNS disease presentations. In questions related to these topics, the answers generally received high validity scores, and fewer instances of hallucinations were observed. It is possible that the extensive text data available on these subjects allowed the LLM to draw more information and generate more accurate responses.

### Patient Safety Concerns in the Answers

Hallucinations may present benign or harmful misinformation, with significant implications in the field of medicine. Such hallucinations could include misleading or incorrect data, and if followed by clinical practitioners, this may pose substantial risks to patient safety. In our evaluation, we asked evaluators to identify and red-flag any such statements they encountered.

Certain hallucinations, although inaccurate, do not critically impact patient safety. For instance, ChatGPT occasionally uses very outdated terminology. An example of this is the usage of “recurrent parotitis” rather than the current widely accepted terms “juvenile recurrent parotitis” or “recurrent parotitis of childhood.”

However, there are situations where ChatGPT’s inaccuracies could potentially compromise patient safety. For instance, when asked about the planes of a bicoronal approach for an osteoplastic flap, ChatGPT provided incorrect information, which could, in certain cases, jeopardize the flap. Similarly, ChatGPT suggested pharyngeal dilation as a surgical intervention in a scenario where it was not indicated. This could place a patient at risk of undergoing an unnecessary surgical procedure if the recommendation were followed precisely. Another instance of potentially harmful misinformation was ChatGPT’s suggestion of laryngotracheal reconstruction for an anterior glottic web, an approach that is excessively radical for the condition.

### The Overall Accuracy of the Results

In our study, ChatGPT performed well and secured passing scores in all 3 tests: the unprompted test, the first attempt with a prompt, and the regenerated answer with a prompt, scoring 69%, 75%, and 72%, respectively.

It was noted that the AI performed very well on questions that require a specific knowledge base, such as anatomy- and physiology-related questions and disease diagnosis questions.

Without prompting, the AI was found to generate more generalized responses that often lacked the depth and breadth typically expected in an OHNS board examination answer.

ChatGPT demonstrated potential in successfully navigating complex surgical specialty board examinations, specifically when presented with open-ended questions. Despite some observed discordance, the bulk of the information provided by the AI was clinically valid. Such features may prove highly beneficial for medical education, such as in equitable access to resources, particularly in low-resource settings where access to such information may not be readily available. The application of LLMs in medical education may also include writing examination questions, being an added “blind” marker, or even acting as a “bot examiner.” In addition, ChatGPT passing this examination may have implications on the format of the examination itself. Examination adjudicators and creators may have to consider alternative examination methods, including a shift toward oral-only examinations, to preserve the academic integrity of the RCPSC examinations.

Some inaccuracies identified were due to the use of outdated data. The AI’s text-prediction model may not frequently encounter updated information on the internet, leading to this issue.

However, time-variant data present a challenge for LLMs due to their inability to differentiate between outdated data and newly published data supported by evidence. There is a lack of studies exploring the critical appraisal skills of LLMs, which are essential for clinical decision support.

Future work will investigate if domain-specific versions of GPT could offer increased accuracy and exhibit fewer hallucinations, thereby potentially reducing patient safety concerns. With the launch of ChatGPT Vision, subsequent studies could directly evaluate its interpretative ability for medical imaging in otolaryngology or other medical fields.

### Limitations

While this study presents valuable insights into the performance of ChatGPT in open-ended OHNS questions, its inherent limitations must also be acknowledged. First, image-based questions could not be used for assessment due to the limitations of the currently available version of ChatGPT, which is based on GPT-4; the public version did not support visual data queries at the time of our test. Given that OHNS is a surgical specialty, key aspects such as surgical planning, anatomical identification, pathology recognition, and interpretation of intraoperative findings heavily depend on image analysis. Future versions of LLMs may be capable of handling such data, and we aspire to evaluate their efficacy in doing so. Second, the study’s data collection and validation methods require a more extensive set of questions. Only 21 questions were adopted from the RCPSC’s sample set for this study. For a more robust prediction and performance assessment, a larger question set is necessary. Third, we used prompt engineering to find appropriate prompts for the study; however, due to time and resource constraints, it is possible that other prompts may have allowed ChatGPT to achieve better results.

### Conclusions

We evaluated the performance of ChatGPT by using it on a sample board-certifying examination of the RCPSC for OHNS, using our novel CVSC framework. ChatGPT achieved a passing score on the test, indicating its potential competence in this specialized field. Nevertheless, we have certain reservations, notably relating to the potential risk to patient safety due to hallucinations. Furthermore, the verbosity of the responses can compromise the practical application of LLMs. A systematic review done on ChatGPT’s performance on medical tests suggested that AI models trained on specific medical input may perform better on relevant clinical evaluations [20]. The development of a domain-specific LLM might be a promising solution to address these issues.

## Appendix

Multimedia Appendix 1 Sample questions from past examinations of the Royal College of Physicians and Surgeons.

Multimedia Appendix 2 Questions and ChatGPT answers (A1).

Multimedia Appendix 3 Questions and ChatGPT answers (A2a).

Multimedia Appendix 4 Questions and ChatGPT answers (A2b).

## Abbreviations

ABNS: American Board of Neurological Surgery
AI: artificial intelligence
AP: Advanced Placement
CVSC: Concordance, Validity, Safety, Competency
GRE: Graduate Record Examinations
LLM: large language model
MCAT: Medical College Admission Test
NCCN: National Comprehensive Cancer Network
OHNS: otolaryngology/head and neck surgery
OKAP: Ophthalmic Knowledge Assessment Program
RCPSC: Royal College of Physicians and Surgeons of Canada
SAT: Scholastic Assessment Test
USMLE: United States Medical Licensing Examination

## References

[1] Varanasi L (2023). AI models like ChatGPT and GPT-4 are acing everything from the bar exam to AP Biology. Here's a list of difficult exams both AI versions have passed. Business Insider.

[2] Kung TH, Cheatham M, Medenilla A, Sillos C, De Leon L, Elepaño Camille, Madriaga M, Aggabao R, Diaz-Candido G, Maningo J, Tseng V (2023). Performance of ChatGPT on USMLE: Potential for AI-assisted medical education using large language models. PLOS Digit Health.

[3] Bommineni V, Bhagwagar S, Balcarcel D, Davazitkos C, Boyer D Performance of ChatGPT on the MCAT: The road to personalized and equitable premedical learning. medrXiv.

[4] Sallam M The utility of ChatGPT as an example of large language models in healthcare education, research and practice: Systematic review on the future perspectives and potential limitations. MedrXiv.

[5] Rudolph J, Tan S, Tan S (2023). ChatGPT: Bullshit spewer or the end of traditional assessments in higher education?. J Appl Med Teach.

[6] Chen S, Kann B, Foote M, Aerts H, Savova G, Mak R, Bitterman D The utility of ChatGPT for cancer treatment information. MedrXiv.

[7] Antaki F, Touma S, Milad D, El-Khoury J, Duval R (2023). Evaluating the performance of ChatGPT in ophthalmology: an analysis of its successes and shortcomings. Ophthalmol Sci.

[8] Sinha RK, Deb Roy A, Kumar N, Mondal H (2023). Applicability of ChatGPT in assisting to solve higher order problems in pathology. Cureus.

[9] Ali R, Tang O, Connolly I, Zadnik SP, Shin J, Fridley J, Asaad W, Cielo D, Oyelese A, Doberstein C, Gokaslan Z, Telfeian A (2023). Performance of ChatGPT and GPT-4 on neurosurgery written board examinations. bioRxiv. Posted online March 25, 2023.

[10] Ali R, Tang O, Connolly I, Fridley J, Shin J Performance of ChatGPT, GPT-4, and Google Bard on a neurosurgery oral boards preparation question bank. MedrXiv.

[11] Nógrádi B, Polgár T, Meszlényi V, Kádár Z, Hertelendy P, Csáti A, Szpisjak L, Halmi D, Erdélyi-Furka B, Tóth M, Molnár F, Tóth D, B?sze Z, Klivényi P, Siklós L, Patai R ChatGPT M.D.: is there any room for generative AI in neurology and other medical areas?. Preprints with The Lancet.

[12] Gilson A, Safranek CW, Huang T, Socrates V, Chi L, Taylor RA, Chartash D (2023). How does ChatGPT perform on the United States Medical Licensing Examination? the implications of large language models for medical education and knowledge assessment. JMIR Med Educ.

[13] Nastasi A, Courtright K, Halpern S, Weissman G Does ChatGPT provide appropriate and equitable medical advice?: A vignette-based, clinical evaluation across care contexts. MedrXiv.

[14] Lee P, Bubeck S, Petro J (2023). Benefits, limits, and risks of GPT-4 as an AI chatbot for medicine. N Engl J Med.

[15] Eysenbach G (2023). The role of ChatGPT, generative language models, and artificial intelligence in medical education: a conversation with chatgpt and a call for papersgenerative language models, and artificial intelligence in medical education: a conversation with ChatGPT and a call for papers. JMIR Med Educ.

[16] Format of the examination in vascular surgery. Royal College of Physicians and Surgeons of Canada.

[17] Zakka C, Chaurasia A, Shad R, Dalal AR, Kim JL, Moor M, Alexander K, Ashley E, Boyd J, Boyd K, Hirsch K, Langlotz C, Nelson J, Hiesinger W (2023). Almanac: Retrieval-augmented language models for clinical medicine. Res Sq.

[18] GPT-4 is OpenAI’s most advanced system, producing safer and more useful responses. OpenAI.

[19] Nori H, King N, McKinney S, Carignan D, Horvitz E Capabilities of GPT-4 on medical challenge problems. ArXiv.

[20] Li J, Dada A, Kleesiek J, Egger J ChatGPT in healthcare: a taxonomy and systematic review. MedrXiv.

